# Biodegradable Polydepsipeptides

**DOI:** 10.3390/ijms10020589

**Published:** 2009-02-13

**Authors:** Yakai Feng, Jintang Guo

**Affiliations:** School of Chemical Engineering and Technology, Tianjin University / Weijin Road 92, 300072 Tianjin, P.R. China

**Keywords:** Polydepsipeptides, morpholine-2, 5-dione, biomaterials, biodegradation, ring-opening polymerization

## Abstract

This paper reviews the synthesis, characterization, biodegradation and usage of bioresorbable polymers based on polydepsipeptides. The ring-opening polymerization of morpholine-2,5-dione derivatives using organic Sn and enzyme lipase is discussed. The dependence of the macroscopic properties of the block copolymers on their structure is also presented. Bioresorbable polymers based on polydepsipeptides could be used as biomaterials in drug controlled release, tissue engineering scaffolding and shape-memory materials.

## 1. Introduction

Synthetic biodegradable polymeric materials are often used for biomedical applications such as surgical sutures, resorbable bone plates, artificial skin, tissue engineering scaffolds, and carrier systems for the controlled release of drugs and genes [1,2]. These materials have been thoroughly investigated and discussed in several review papers [3–5]. The requirements in the selection of any biomedical material, beside biodegradability and resorbability, are that it should have suitable mechanical properties and not produce toxic degradation products.

Two important classes currently investigated for a wide variety of surgical and pharmaceutical applications are poly(α-hydroxy acid)s, poly(α-amino acid)s and their block copolymers [6,7]. The synthesis of poly(α-hydroxy acid)s has been extensively studied and reported. The most important method for the synthesis of biodegradable polyesters is the ring-opening polymerization of the corresponding cyclic mono- or diesters. Poly(glycolic acid) (PGA), poly(l-lactic acid) (PLLA), poly(d,l-lactic acid) (PDLLA) are synthesized by ring-opening polymerization of the corresponding six-membered cyclic diesters, i.e., glycolide, l-lactide and d,l-lactide. PGA and PLLA are semicrystalline polymers, whereas PDLLA is amorphous. PGA has a glass transition temperature (T_g_) close to body temperature (T_g_ = 36 °C), whereas PLLA and PDLLA have T_g_ values above body temperature (T_g_ = 57 – 60 °C and T_g_ = 50 – 54 °C, respectively) [8,9]. PLLA is one of the most widely used biodegradable polymers, with a variety of biomedical and pharmaceutical applications. PLLA has excellent mechanical properties and can be slowly broken down into nontoxic metabolites. However, its applications have been somewhat limited because of difficulties in controlling the hydrolysis rate, poor hydrophilicity, and high rigidity and crystallinity [10–12]. Poly(ε-caprolactone) (PCL) is relatively nontoxic, biocompatibility, and possesses sufficient mechanical strength and thermal stability for scaffolding applications. However, a major limitation in the application of PCL is the slow degradation rate due to its characterized high hydrophobicity and crystallinity [13].

Since the properties of homopolymers are more or less fixed, copolymers are required to provide materials with a wide range of properties. In 1979, Gilding reported that copolymers of lactic acid and glycolic acid (GA) showed a range of properties, depending on their composition [14].

Polypeptides for biomedical uses are classified into two categories: natural and synthetic polypeptides. The former includes, for example, collagen, gelatin, and other water-soluble proteins. Since proteins are composed of amino acids, many researchers have tried to develop synthetic polypeptides derived from amino acids to serve as models for structural, biological, and immunological studies. In addition, many different types of synthetic polypeptides have been investigated for biomedical applications [15]. Synthetic polypeptides have several potential advantages as biomaterials. Many types of synthetic polypeptides have been prepared for biomedical applications, such as sutures, artificial skin substrates, and drug delivery systems [16–18]. Side chains offer sites for the attachment of drugs, crosslinking agents, or pendant groups that can be used to modify the physico-chemical properties of the polymer. Because these polymers release naturally occurring amino acids as the primary products of polymer backbone cleavage, their degradation products may be expected to show a low level of systemic toxicity. In spite of their apparent potential as biomaterials, synthetic polypeptides have actually shown few practical applications. Most synthetic polypeptides are highly insoluble and non-processable materials. Owing to these difficulties, only a few synthetic polypeptides, usually derivatives of poly((*S*)-glutamic acid) are currently investigated as implant materials.

Copolymers of α-hydroxy acids and α-amino acids, which are called polydepsipeptides, are a valuable addition to the existing list of synthetic biodegradable polymers [19]. Since these polymers contain both ester and amide groups in the chain, their biodegradation behavior is different from that of the homopolymers. Linear polydepsipeptides were initially produced by stepwise active ester-peptide coupling reactions [20–29]. Ring-opening polymerization of morpholine-2,5-dione derivatives (cyclic dimers of α-hydroxy- and α-amino acid) is the second and more attractive way to produce these poly(ester amide)s.

## 2. Synthesis of morpholine-2,5-dione derivatives

**Scheme 1:**
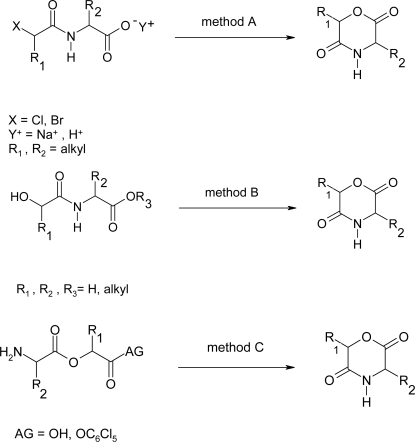
Synthesis methods of morpholine-2,5-dione derivatives.

Morpholine-2,5-dione derivatives, which have a ester group and a amide group in one 6-membered ring, are formally cyclic dimers of an α-hydroxyacid and an α-amino acid. Morpholine-2,5-dione derivatives are the simplest cyclic depsipeptides. Morpholine-2,5-diones play a role as intermediates in the synthesis of optic active α-amino acids, furthermore, they act as monomers for synthesis of polydepsipeptides and their random copolymers or block copolymers with high molecular weight. Three general methods to synthesize morpholine-2,5-dione derivatives have been reported (Scheme 1).

### 2.1. Method A

Chadwick and Pascu reported on the synthesis of 6-methylmorpholine-2,5-dione via heating of *N*-(2-bromopropionyl)-glycine sodium salt [30]. Many morpholine-2,5-diones have been synthesized by this method [31–34]. The *N*-(α-haloacyl)-α-amino acids and *N*-(α-halopropionyl)-α-amino acids used as starting materials were prepared in high yields by acylation of amino acids with bromo-propionyl bromide or chloroacetyl chloride under Schotten Baumann reaction conditions. Kricheldorf synthesized *N*-chloroacetyl amino acids in another way whereby α-amino acids were silylated with chlorotrimethylsilane and triethylamine, and the silylated amino acids were reacted with chloroacetyl chloride, a clean and a nearly quantitative reaction [35]. The silyl esters were hydrolyzed and directly transformed into the corresponding sodium salts. They tried to synthesize morpholine-2,5-diones directly from silyl esters using benzyltriethylammonium chloride as catalyst, but only obtained brownish oils or black tars.

The cyclization of *N*-(α-haloacyl)-α-amino acids and N-(α-halopropionyl)-α-amino acids was carried out in high diluted solution of dimethylformamide with triethylamine, NaOH, NaCO_3_, or NaHCO_3_ to neutralize the acid products. During the cyclization reaction of optically active morpholine-2,5-diones, racemisation at C6 of the morpholine-2,5-diones was found. The cyclization reaction goes through an intramolecular S_N_ reaction mechanism, rather than via a S_N1_ or via a S_N2_. Besides racemisation, intermolecular reaction, as the main observed side reaction, produced linear oligomers. These competing reactions decreased the yields of morpholine-2,5-diones. However, morpholine-2,5-diones with protected functional groups were synthesized via this method with 38–55% yield.

### 2.2. Method B

Hartwig and Schoellkopf first reported the synthesis of morpholine-2,5-diones according to Method B [36]. The cyclization of *N*-(α-hydroxyacyl)- α-amino acids was carried out in high yield in a highly diluted solution of chloroform and benzene. The cyclization reaction involves an intramolecular esterification mechanism, which should not cause racemisation at C6 of morpholine-2,5-diones, because no nucleophilic attack takes place at the C6 stereocenter during the cyclization, but (3*S*,6*RS*)-dibenzylmorpholine-2,5-dione was partially racemised at the C6 stereocenter, although it is not clear whether the racemisation took place during the cyclization reaction or in the other synthesis steps preceeding the cyclization reaction. Nakamura synthesized morpholine-2,5-diones via trans-esterification of *N*-(α-hydroxyacyl)- α-amino acids esters in toluene at 150 – 350 °C. The high reaction temperature resulted in racemisation of the α-amino acids esters [37].

The cyclization of *N*-(α-hydroxyacyl)-α-amino acids was usually performed in three different ways [38]. The acid catalyzed reaction using *p*-toluenesulfonic acid, methanesulfonic acid or trifluoromethanesulfonic acid as catalyst gave the cyclized morpholine-2,5-dione products in 38% yield. Cyclization by carbonyl diimidazole was quantitative, however the separation of morpholine-2,5-dione from imidazole was tedious and resulted in overall low yields. Under reduced pressure and high temperature *N*-(α-hydroxyacyl)- α-amino acids were most simply cyclized spontaneously to form morpholine-2,5-diones, along with some oligomers.

### 2.3. Method C

(3*S*,6*S*)-3,6-Diisopropylmorpholine-2,5-dione and (3*S*,6*S*)-3-isobutyl-6-isopropyl-morpholine-2,5-dione were synthesized via Method C by cyclization of *O*-(α-aminoacyl)-α-hydroxycarboxylic acids. Goodmann *et al*. reported that during cyclization the trifluoracetic salt of *O*-(α-aminoacyl)-1-methyl-2-oxo-2-(pentachlorophenoxy)ethyl ester is polycondensated to oligomers besides giving morpholine-2,5-diones as main products [39]. In’t Veld synthesized morpholine-2,5-diones in low yields (4%–10%) according to Method C [40]. Racemisation at C6 and C3 of the morpholine-2,5-diones was not observed during the cyclization reaction. This method involves a multi-step organic synthesis, similar to peptide chemistry. The yield of all involved synthesis steps was lower than 10%.

The carboxylic acid group of chloroacetyl chloride was protected with polystyrene resin containing indole functional groups to form an intermediate which then reacted with BocNHCH(CH_2_C_6_H_5_)CO_2_H, BocNHCH(Me)CO_2_H, and BocNHCH(CH_2_CH(Me)_2_)CO_2_H in the presence of Cs_2_CO_3_/*n*-Bu_4_NI to afford the corresponding conjugate resins [41]. After treatment with trifluoroacetic acid to deprotect the Boc groups, the resins were irradiated with UV light leading to the corresponding morpholine-2,5-diones in high yields and purities via intramolecular photo-induced cyclorelease.

**Scheme 2:**
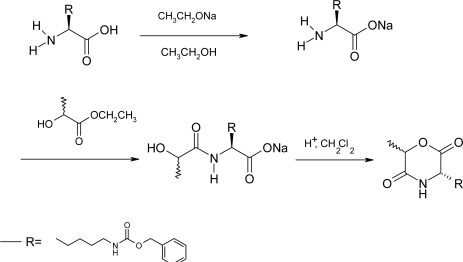
Reaction scheme of the preparation of Cyclo(DLLA-Lys(Z)).

**Scheme 3:**
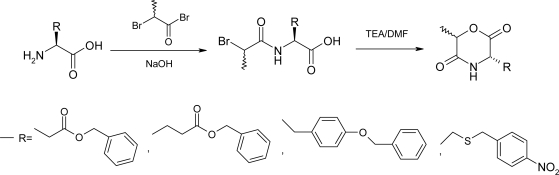
Reaction scheme of the preparation of Cyclo(DLLA-Asp(OBzl)), Cyclo(DLLA-Glu(OBzl)), Cyclo(DLLA-Tyr(Bzl)) and Cyclo(DLLA-Cys(*p*NBzl)).

The synthesis of morpholine-2,5-diones with pendant functional groups, i.e. Cyclo[DLLA-Asp(OBzl)], Cyclo[DLLA-Glu(OBzl)], Cyclo[DLLA-Lys(Z)], Cyclo[DLLA-Cys(*p*NBzl)], Cyclo-[DLLA-Tyr(Bzl)] was performed as shown in Schemes 2 and 3.

In order to avoid side reactions, the pendant functional groups of the trifunctional amino acid residues had to be temporarily blocked by protecting groups which are stable to the projected reactions outlined in Scheme 2 and 3. Moreover, the protective groups have to be stable during the ring-opening polymerization of the morpholine-2,5-diones. Subsequently the protective groups must be selectively removed without cleavage of ester and/or amide bonds of the polymer main chains. On the basis of literature the benzyl ester was selected as the protective group for the β- and γ-carboxylic acid function of Asp and Glu, respectively [42]. The benzyl group was also used as a protective group for the hydroxy function of Tyr. The ε-amino group of the Lys was protected by the benzyloxycarbonyl group. Feijen *et al*. [42] reported the *p*-methoxybenzyl group was used as protective group for Cys. However, the removal of the *p*-methoxybenzyl group was performed with trifluoromethanesulfonic acid. The deprotection condition might cause ester and/or amide bond cleavage in the polymer main chain. The molecular weight of the polymer decreases extremely after deprotection. The *p*-nitrobenzyl group was also used as the protective group for thiol group of Cys. The protective groups could easily be removed by means of catalytic hydrogenation.

Cyclo[DLLA-Lys(Z)] was prepared in 25% yield by intramolecular cyclization of *N*^α^-2(*R,S*)-[hydroxypropionyl]-*N*^ε^-(benzyloxylcarbonyl)-(*S*)-lysine. Attempts to synthesize Cyclo[DLLA-Asp(OBzl)] and Cyclo[DLLA-Glu(OBzl)] using similar methods failed. Most probably transesterification reactions at the protective benzyl ester groups of β-benzyl-*N*-[2(*R,S*)-hydroxy-propionyl]-(*S*)-aspartate and γ-benzyl-*N*-[2(*R,S*)-hydroxypropionyl]-(*S*)-glutamate occur during the cyclization procedure. Cyclo[DLLA-Asp(OBzl)], Cyclo[DLLA-Glu(OBzl)] and Cyclo[DLLA-Tyr(Bzl)] were synthesized according to Scheme 3 with TEA in a large amount of DMF for 3 h at 100°C. Purification of the obtained morpholine-2,5-diones with functional groups was generally carried out by column chromatography on silica gel using ethyl acetate as an eluent, followed by recrystallization from ethyl acetate/hexane (1:3 v/v), although purified Cyclo[DLLA-Asp(OBzl)] was obtained by crystallization of the reaction mixture from toluene without column chromatography. The yield of the protected morpholine-2,5-diones was low. It is suggested that the intramolecular cyclization occurs in competition with the intermolecular reaction even at high dilution.

Cyclo[DLLA-Cys(*p*NBzl)] was prepared by treatment of *N*-[2(*R,S*)-bromopropionyl]-*S*-*p*-nitrobenzyl-(*R*)-cysteine with TEA in DMF for 17 h at room temperature. When the reaction was carried out at 100 °C for 3 h, partial deprotection of the thiol group resulted in the formation of several side products. Yao synthesized Cyclo[DLLA-Lys(Z)] via intramolecular cyclization of *N*^α^-[-bromoacetyl]-*N*^ε^-(benzyloxylcarbonyl)-(*S*)-lysine with TEA in DMF for 24 h at 60°C with 66% yield [43,44]. However, Cyclo[DLLA-Lys(Z)] contained trace Br impurities, and should be further purified by choosing suitable solvents to improve the purity for ring-opening polymerization.

## 3. Ring-opening polymerization of morpholine-2,5-dione derivatives

### 3.1. Ring-opening polymerization of morpholine-2,5-dione derivatives using organic Sn as catalyst

**Scheme 4:**
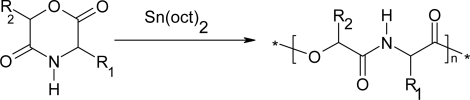
Ring-opening polymerization of different morpholine-2,5-dione.

In 1985, Helder *et al*. synthesized for the first time a polydepsipeptide by ring-opening polymerization of morpholine-2,5-diones [45]. The polymerization was carried out in the bulk at 130°C for 28 to 48h using stannous octoate [Sn(Oct)_2_] as a catalyst. Many researchers have subsequently studied the ring-opening polymerization (Shown in Scheme 4) of different morpholine-2,5-dione derivatives [46–48].

Sn(Oct)_2_ is typically used in melt polymerization of morpholine-2,5-diones and is tolerant to water and added alcohols. The mechanism of polymerization is generally agreed that the initiating group is a tin(II) hydroxyl or alkoxide formed reversibly in the reaction:

$$\text{Sn}(\text{Oct}{)}_{2}+{\text{HX}}_{1}⇌(\text{Oct}){\text{SnX}}_{1}+\text{HOct}$$

where X_1_ = OH or OR. Water or an alcohol is thus a necessary initiator, and Sn(Oct)_2_ acts as catalyst.

When the molar amount of Sn(Oct)_2_ was similar to that of alcohol initiators, the molecular weight of the obtained polymorpholine-2,5-diones increased with the increasing molar ratio of monomer to initiator, and conversion of the monomer without 6-substituents. However, morpholine-2,5-diones with 6-substituent groups showed very low ring-opening polymerization reactivity compared with lactide. It was not possible to explain the decrease of reactivity of the monomers considering only the steric hindrance of 6-substituent groups in morpholine-2,5-diones Especially, *N*-alkyl substituted morpholine-2,5-diones could not take place the ring-opening polymerization to obtain the polydepsipeptides with *N*-alkyl substituted α-amino acid residues, such as poly(*N*-methylglycine-alt-DLLA) and poly(*N*-isopropylglycine-alt-DLLA) [49]. This indicates that the amido N-H group in the monomers may be involved in a reaction leading to a chelating intermediate when ring-opening occurred by attack at the ester group [50].

Kricheldorf *et al*. investigated the ring-opening polymerization of morpholine-2,5-dione and 3*R,S*-methylmorpholine-2,5-dione with 2,2-dibutyl-2-stanna-1,3-dioxepane (DSDOP) as initiator with different times and monomer/initiator (M/I)-ratios [51]. The DSDOP initiated polymerizations gave slightly higher molecular weights than the polymers obtained using Sn(Oct)_2_, but the molecular weights were rather low in all cases and was not proportional to the M/I-ratio.

The first successful copolymerizations were performed with *p*-dioxanone and unsubstituted or alkyl-substituted morpholine-2,5-diones [52]. These copolymers showed *in vivo* accelerated resorption with excellent strength retention. Yonezawa synthesized copolymers of 6-isopropylmorpholine-2,5-dione or 6-isopropyl-3-methylmorpholine-2,5-dione with DLLA; however, polymer yields and viscosities were rather low and the incorporation of depsipeptide units was extremely low [53].

Alternating poly(glycine-d,l-lactic acid) was obtained from 6-methylmorpholine-2,5-dione by ring-opening polymerization, and random copolymers were also synthesized by copolymerization of morpholine-2,5-diones with DLLA [54]. Some polydepsipeptides such as poly(glycolic acid-alanine-d,l-lactic acid) and poly(d,l-lactic acid-alanine) were obtained by ring-opening copolymerization of 3-methyl- and 3,6-dimethylmorpholine-2,5-dione, respectively, with DLLA [55]. In 1989, Fung synthesized bioresorbable polydepsipeptides for medical implant devices from 3-alkyl-substituted-morpholine-2,5-dione [56]. Copolymerizations of ε-caprolactone (CL) with several morpholine-2,5-dione derivatives were investigated by In′t Veld and associates [33].

In order to improve the hydrophilicity of polydepsipeptides, one may introduce hydrophilic pendant functional groups into the polymer chain. Feijen *et al*. reported the copolymerization of CL and morpholine-2,5-dione with pendant hydrophilic functional groups [33,42,57]. However, they failed to achieve copolymers with a content of functional monomer exceeding 20%. The ε-amino groups in linear poly(l-lactic acid-*co*-*S*-lysine) with about 1% lysine content have been utilized to initiate the ring-opening polymerization of the amino acid N-carboxyanhydrides to yield graft copolymers [58]. However, the reaction process is very complicated. Ouchi *et al*. reported the synthesis of cyclo(Glc-Asp(OBzl)) and its homopolymer [59]. The molecular weight of the resulting polymer ranges from 1,950 to 3,280. Wang *et al*. synthesized a polymer with high molecular weight ( ${\bar{M}}_{n}=5,800-13,500$) via homopolymerization using stannous octoate as a catalyst [31,32]. The IR spectrum of Wang’s monomer is rather different from that of Ouchi’s in the range of the N-H stretch band [31,32]. This difference may indicate the existence of different association forms of the monomer on the basis of the hydrogen bond. The hydrogen bonding in the monomer has a large effect on its polymerization reactivity. Selection of a suitable recrystallization solvent system results in different types of monomer crystals in which either intramolecular or intermolecular hydrogen bonds are formed. This helps to achieve a higher reactivity in the ring-opening polymerization.

Feijen *et al*. and Barrera *et al*. reported that the ring-opening homopolymerization of 3-methyl-morpholine-2,5-dione derivatives with protected functional substituents was not successful because of the low reactivity of the monomers [42,60]. However, Ouchi *et al*. synthesized three kinds of protected polydepsipeptides having number-average degrees of polymerization of 9–13 from morpholine-2,5-dione derivatives with glycolic acid (Glc) and l-Lys, l-Asp or l-Glu [61]. This indicates the difference in the polymerization reactivity between morpholine-2,5-dione derivatives with Lac residue and with Glc residue because the 3-methyl-substituted group in morpholine-2,5-dione derivatives has higher steric hindrance than a H atom at the 3 position. Morpholine-2,5-dione derivatives without 3-alkyl-substituted groups showed higher polymerizability in homopolymerization and copolymerization with other cyclomonomers.

The biodegradable l-lactic acid-depsipeptide copolymers having pendant reactive groups, poly[LLA-(Glc-Asp)], poly(LLA-Lys) and poly[LLA-(Glc-Lys)], were obtained by ring-opening polymerization of LLA with cyclodepsipeptides consisting of Glc and Asp or Lys [60,61]. The polymers showed enzymatic degradation and also fast degradation rates without the presence of enzymes, when compared with PLLA [62]. Ouchi *et al*. reported the preparation of biodegradable microspheres from poly[LLA-(Glc-Asp)] and poly[LLA-(Glc-Lys)] with low contents of depsipeptide units by the oil-in-water/solvent evaporation method [62]. The average diameters of the dried microspheres were estimated to be 390–450 nm by scanning electron microscopy. The amount of functionalized groups located on the surfaces increased with increasing content of depsipeptide units in the copolymers used. John synthesized biodegradable cross-linked microspheres from poly[CL-(Glc-Ser)] [63]. Poly[CL-(Glc-Ser)] was prepared by copolymerization of CL and Cyclo[Glc-Ser(OBzl)]. After deprotection, the pendant OH groups of the serine residue were acrylated. Cross-linked beads of ca. 25–170 μm size were obtained by UV-suspension polymerization. The beads are opaque after swelling in water and transparent in DMSO and CHCl_3_, and may be useful as biodegradable microspheres for drug delivery.

### 3.2. Ring-opening polymerization of morpholine-2,5-dione derivatives using CaH_2_ as catalyst

**Scheme 5:**
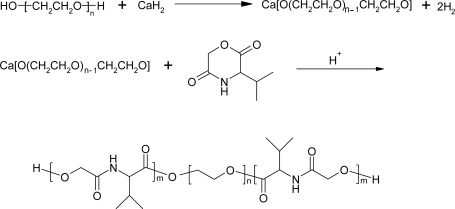
Reaction mechanism of CaH_2_ as a co-initiator for the ring-opening polymerization of 3(S)-isopropyl-morpholine-2,5-dione in the presence of PEO.

Feng *et al*. reported on biodegradable ABA triblock copolymers, with A being a poly(glycolic acid-valine) block and B being a PEO block [64]. These block copolymers were prepared by ring-opening polymerization of cyclo(glycolic acid-valine) using Ca-alcoholates of hydroxytelechelic PEO as the initiator (Scheme 5). Residual Ca compounds in the final product are expected to be nontoxic.

**Scheme 6:**
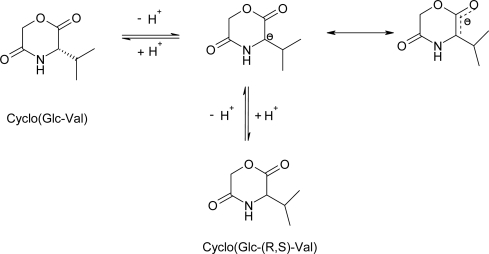
Racemization mechanism of Cyclo(Glc-Val) during the polymerization.

Block copolymers were synthesized in yields ranging from 37 to 61% by ring-opening polymerization of cyclo(glycolic acid-valine) with an initiator system comprising hydroxytelechelic PEO6000 and CaH_2_ at 140°C for 24 or 96 h. The polymer yield obtained with Ca-alcoholate as initiator at 140°C for 24 h is generally lower than that obtained using Sn(Oct) _2_ as a catalyst at 140°C for 9 h. The racemization of the l-valine residue during copolymerization of cyclo(glycolic acid-valine) with PEO in the presence of CaH_2_ was confirmed by ^1^H-NMR. More than 40% of the l-valine residues were racemized at 140°C within 96 h, while about 12% were racemized within 24 h. Cyclo(glycolic acid-valine) was sensitive to racemization because after proton abstraction the negative charge in the cyclic molecule is delocalized (Scheme 6). Potassium ethoxide (KOEt) was used as an initiator in the anionic ring-opening polymerization of protected morpholine-2,5-diones to prepare polydepsipeptides as macroinitiators for synthesis of diblock copolymers, however, the optical activity of the polydepsipeptides was not investigated [65].

### 3.2. Ring-opening polymerization of morpholine-2,5-dione derivatives using enzymes as catalyst

The polymer industries rely heavily on inorganic catalysts for synthesis and processing, and some of these catalysts are prepared from toxic and/or precious metals. Negative environmental impact has been a concern raised by use of many of these metals, and recently more emphasis is being given to green technology, including the use of nontoxic catalysts such as enzymes in materials processing. Enzymes are increasingly being used as catalysts in testing, evaluation, processing, and production in chemical, food, pharmaceutical, agricultural, and health care industries.

During the past decade, studies have shown that enzyme-catalyzed reactions also can be carried out in anhydrous organic solvents and solvents with differing amounts of water [66]. It turns out that water is not the ideal medium in some enzyme-catalyzed reactions for the following reasons: stereoselectivity and regioselectivity of reactions, new reactions with favorable specificity and thermodynamic equilibrium, improved solubility of hydrophobic monomers, oligomers, and polymers, control of molecular weight and dispersity of polymers, mild or ambient operating conditions such as pH, temperature, and pressure; improved stability of the enzyme, and control of microbial contamination in the medium. From a processing point of view, ease of separation and reuse of enzymes and separation of products from the reaction medium are additional advantages of non-aqueous enzyme-based reactions.

Enzyme-catalyzed polymerization (enzymatic polymerization) has attracted much attention as a new method of polymer synthesis [67–70]. Specific enzymatic catalysis is expected to synthesize polymers with high selectivity or with novel structures. The term “enzymatic polymerization” is always defined as a polymerization *in vitro* via a nonbiosynthetic pathway catalyzed by an isolated enzyme. Until now, there have been many papers on enzymatic syntheses of natural biopolymers as well as non-natural synthetic polymers.

The ring-opening polymerization of morphoilne-2,5-diones were carried out in the presence of enzymes, such as lipases [71,72]. The effects of enzymes, reaction temperature, reaction time and water content in the reaction mixtures on monomer conversion and product molecular weight were investigated in literatures. Although Novo-435 was efficient in catalyzing the ring-opening polymerization of ε-caprolactone at 60°C [73], the heat stability of Novo-435 is very poor. Therefore it failed to polymerize the cyclic depsipeptide. Lipase PPL was mainly employed as a catalyst yielding morphoilne-2,5-diones with high molecular weight and high yields (Table 1).

The configuration of the amino acid moieties, i.e. *R-, S-*, or *R,S-*, in the monomers did not affect the enzyme-catalyzed ring-opening polymerization of morpholine-2,5-diones [74]. The conversion of morpholine-2,5-diones decreased significantly when methyl substituents were introduced into the hydroxyl acid residues. The configuration of the lactic acid residue in the monomer strongly affected the polymerization behavior. The reason may be that the enzyme-catalyzed polymerization of morpholine-2,5-dione derivatives takes place at the ester group of the morpholine-2,5-dione derivatives and that the steric effect of the methyl group decreases the activity of the monomers. Further, PPL might favor the polymerization of 6(*S*)-methyl-morpholine-2,5-dione compared with 6(*R,S*)-methyl-morpholine-2,5-dione [75].

The specific rotations of polydepsipeptdies prepared via PPL catalyzed ring-opening polymerization were significantly smaller than those of polymers prepared via Sn(Oct)_2_-catalyzed polymerization. This is caused by the enhanced racemization of the amino acids residues during enzymatic polymerization. This indicates that *R*-, *S*-amino acids and *S*-lactic acid residues were racemized during PPL catalyzed ring-opening polymerization of morpholine-2,5-diones.

**Scheme 7:**
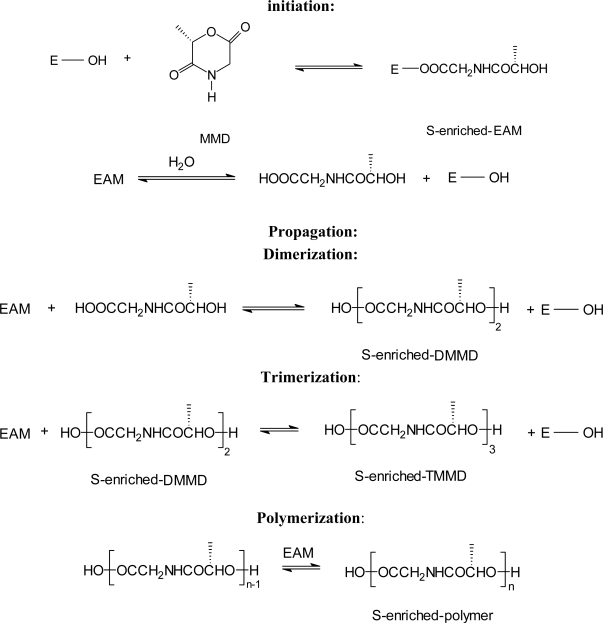
PPL catalyzed polymerization of 6(*S*)-methyl-morpholine-2,5-dione.

The proposed mechanism for lipase-catalyzed ring-opening polymerization of morpholine-2,5-diones is shown in Scheme 7 using 6(*S*)-methylmorpholine-2,5-dione (MMD) as an example. It is based on: (i) polymorpholine-2,5-diones with a carboxylic acid group at one end and a hydroxy group at the other end; (ii) the effect of water on ${\bar{M}}_{n}$ of polymorpholine-2,5-diones; and (iii) mechanisms proposed previous for lipase-catalyzed ring-opening polymerizations of lactones or cyclic carbonates [76–79]. Initiation involves (i) the reaction of morpholine-2,5-diones with the lipase to form the *S*-enriched enzyme-activated-monomer (EAM) complex and (ii) the reaction of EAM with water to form *S*-enriched-*N*-(α-hydroxyacyl)-glycine. Propagation as defined by the presence of an ester function involves the formation of S-enriched-DMMD by reaction of the EAM with *S*-enriched-*N*-(α-hydroxy acyl)-glycine, *S*-enriched-TMMD synthesis by reaction of DMMD with EAM, and subsequent propagation reactions to form high molecular weight chains [72].

**Scheme 8:**
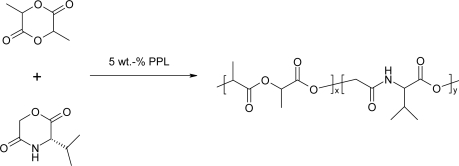
PPL catalyzed ring-opening copolymerization of 3(*S*)-isopropylmorpholine-2,5-dione and DLLA.

The copolymerization of 3(*S*)-isopropylmorpholine-2,5-dione (IPMD) and DLLA was conducted under similar conditions. Copolymers were obtained in the presence of PPL as a catalyst at 100 °C for 168 h (Scheme 8) [80]. While the mole fraction of DLLA in the feed decreased from 100 to 15%, the conversion of DLLA in the copolymerization remained unchanged at about 85%. On the other hand, the conversion of IPMD increased from 10 to 66% while the mole fraction of IPMD in the feed increased from 18 to 100%. The mole fraction of IPMD in the high-molecular-weight fraction of the copolymers increased with increasing mole fraction of IPMD in the feed.

## 4. Biodegradation of polydepsipeptides and their copolymers

Joeress investigated the biodegradation of poly((*S*)Lac-Val) and poly(Glc-Val) *in vitro* at 70 °C [46]. After 357 h, 95% degradation product was *N*-(hydroxylacetyl)-(*S*)-valine, and near 5% resulted from amide bond cleavage. Schakenraad and Helder also found that only ester bonds were hydrolyzed during degradation of polydepsipeptides with various contents of glycine and lactide, the amide bonds were relative stable [81,82]. The rate of degradation of poly(Glc-Val) was 10 times higher than that of poly((*S*)Lac-Val) in PBS solution (pH = 7.4). The high steric hindrance of lactide groups in poly((*S*)Lac-Val) decreased the rate of acid catalyzed hydrolysis of ester bonds. The hydrolytic degradation of polydepsipeptides proceeded probably via bulk hydrolysis, namely the rate of diffusion of the Hydrolytic medium into the polymer matrix is higher than the rate of polymer hydrolysis.

**Scheme 9:**
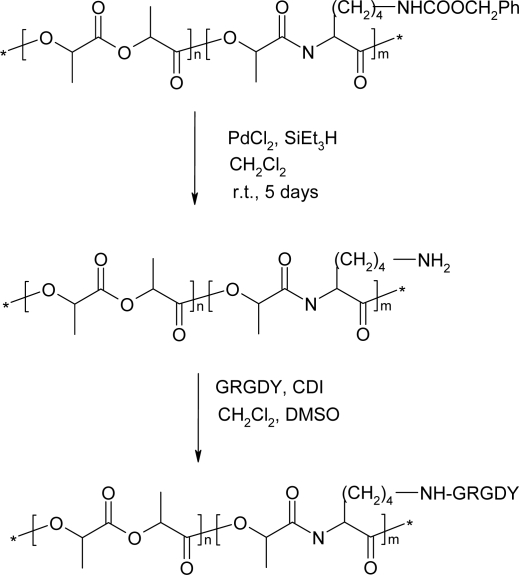
Poly(LA-Lys) with biologically active Arg-Gly-Asp peptide.

Feijen *et al*. reported that 3-alkyl-substituted morpholine-2,5-diones with functionalized side-chain groups were copolymerized with d,l-lactide or ε-caprolactone to afford poly(ester amide)s with pendant functionalized groups [42]. The pendant functionalized groups could affect the hydrophilicity and biodegradability. Langer *et al*. synthesized poly(LA-Lys) with pendant amino functional groups and attached the biologically active Arg-Gly-Asp (RGD) peptide onto the Lys residue (Scheme 9) [83]. Poly(LA-Asp) with carboxylic acid functional groups was synthesized via deprotection of copolymers of (3*S*,6*R,S*)-3-[(benzyloxycarbonyl)methyl]-6-methylmorpholine-2,5-dione with DLLA [84]. The carboxyl functionalized groups were activated and reacted with RGD.

The random copolymers of LA and morpholine-2,5-diones with reactive (hydrophilic) side-chain groups such as poly[(Glc-Asp)-*r*-LA], poly[(Glc-Lys)-*r*-LA], and poly[(Glc-Cys)-*r*-LA], had amphiphilic structures, i.e. hydrophilic side-chain groups and hydrophobic LA main chains [62]. Ouchi *et al*. used these copolymers to prepare microspheres (MSs) with reactive surfaces [62,85]. The MSs obtained by chemical modification of the functional groups with galactose residues could be recognized by a lectin and receptors on hepatoma cells. Moreover, the MSs showed an efficient entrapment of an ionic drug and a slower release of the drug because of the electrostatic interaction of the drug with the ionic side-chain groups in the bulk matrix of the MSs. The surface-modifiable and biodegradable MSs were useful for the cell-specific drug delivery of ionic (water-soluble) drugs.

**Scheme 10:**
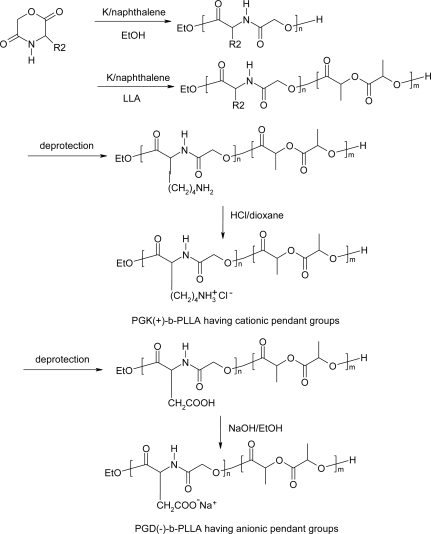
Polydepsipeptide-block-poly(l-lactide)s having amino or carboxylic acid groups.

Polydepsipeptide-block-poly(l-lactide)s having amino or carboxylic acid groups on their side chains were synthesized through anionic ring-opening polymerizations of l-lactide using the corresponding protected polydepsipeptides as macroinitiators and consequent deprotection (Scheme 10). The amphiphilic copolymers consisting of PLLA as hydrophobic segments and polydepsipeptides with amino or carboxylic acid groups as hydrophilic segments were converted to ionic block copolymers PGK(+)-*b*-PLLA having cationic pendant groups and PGD(−)-*b*-PLLA having anionic pendant groups [65]. The ionic block copolymers acted as surfactants preparing poly(L-lactide)-based MSs by an oil-in-water emulsion method. The amount of ionic groups located on the surfaces of the MSs increased with increasing the feed of ionic block copolymers in the polymer blends, whereas the average diameters of the dried MSs decreased.

Poly[depsipeptide-block-(dl-lactide)]s having cationic pendant groups (PGK(+)-*b*-PDLLA) and having anionic pendant groups (PGD(−)-*b*-PDLLA) acted as good biodegradable surfactants to stabilize the corresponding primary water inner phases having proteins in PLGA-based MSs [86]. This led the sustained release of the proteins without initial burst. The PLGA-based MSs prepared using poly(depsipeptide-block-lactide) having ionic pendant groups could be applied as drug release devices for protein drugs. PGK(+)-*b*-PDLLA and PGD(−)-*b*-PDLLA are very interesting biodegradable surfactants from the standpoint of biomedical materials.

Polydepsipeptide networks and cross-linked beads have been prepared by UV photopolymerization of acrylated poly(CL-*co*-GA-*co*-l-serine) and poly(lactic acid-*co*-lysine)-*g*-poly(aspartic acid) [87,88]. The network showed relatively low swelling in water but is easily swelled in chloroform and in dimethyl sulfoxide. The acrylate poly(CL-*co*-GA-*co*-l-serine) was copolymerized with 2-hydroxyethyl methacrylate (HEMA) resulted in cross-linked networks poly(CL-*co*-GA-*co*-l-serine)-HEMA. HEMA improved the swellability in water due to the hydrophilic hydroxyl groups.

Poly(l-lactide-*co*-glycolic acid-*co*-β-benzyl-l-aspartate) [poly(LLA-BMD)] has higher hydrophilicity than PLGA, because amide groups in poly(LLA-BMD) chain improved hydrophilicity of the material and resulted in the increase of water absorption. Poly(LLA-BMD) almost degraded completely in PBS at 37°C in 90 days. The chemical structure of poly(LLA-BMD) was not observed any change during degradation [89]. The molar composition of rest poly(LLA-BMD) materials stayed almost constant along with degradation time, which indicated that LLA and BMD showed proximately degradation rate.

Shirahama investigated the enzymatic degradation of optically active polydepsipeptides based on d-, l-, or dl-alanine [90]. Among the rhizopus delemar lipase, cholesterol esterase (from pseudomonas sp.), and proteinase K (from tritirachium album) used, only proteinase K recognized the isomerism of 3,6-dimethyl-2,5-morpholinedione (DMO), resulting in the following order of degradability: poly[(*L*-DMO-DLLA)-CL] > poly[(dl-DMO-DLLA)-CL] > poly[(d-DMO-DLLA)-CL], i.e. this enzyme has the highest substrate specificity for naturally occurring l-alanine. The enzymatic degradability rate of poly(l-DMO-DLLA) is greater than that of poly(l-DMO-LLA) copolymers with an identical DMO/LA ratio. This is probably due to the greater permeability of water into amorphous poly(l-DMO-DLLA) copolymers than the crystalline poly(l-DMO-LLA).

Ouchi *et al*. studied the enzymatic degradation of polydesipeptides with functionalized side-chain groups using trypsin, V8 protease and papain as enzymes [91]. The polydesipeptides involving amide and ester bonds in the main chain as expected were degraded through competition between the protease action and esterase action. The polydepsipeptides were more easily subjected to hydrolysis by esterase-type enzyme rather than by protease-type enzyme. Ester bonds of the main chain of water-insoluble poly[LA-(Glc-Lys)] and poly[LA-(Glc-Asp)] were cleaved specifically by trypsin and V8 protease, respectively.

The degradation behavior of poly[LA-(Glc-Cys)] with 3.0 mol% of Cys was investigated in KH_2_PO_4_/Na_2_HPO_4_ buffer (pH = 7.0) at 37°C using papain as an enzyme [92]. The main chain bond of poly[LA-(Glc-Cys)] was cleaved slowly in the nonenzymatic degradation system and the degradation rate of this copolymer was accelerated in the presence of papain *in vitro*. The degradation rate of poly[LA-(Glc-Cys)] with papain was faster than that of nonenzymatic degradation. The susceptibility of enzyme to amino acid units was reflected on the specific cleavage of ester bonds of (Glc-Cys) units of the main chain in poly[LA-(Glc-Cys)].

Ohya *et al*. synthesized thermosensitive biodegeradable polymers based on polydepsipeptide. The thermosensitive polydepsipeptide poly[Glc-Asn(N-isopropyl)] could be degraded *in vitro* at room temperature by cleavage of the ester bonds in the main chain [93]. The molecular weight of poly[Glc-Asn(*N*-isopropyl)] decreased to 25% of that of the initial value after 7 d. As expected, the poly[Glc-Asn(*N*-isopropyl)] degraded to a monomer level through cleavage of the ester bonds in the main chain in water. The degradation products of poly[Glc-Asn(*N*-isopropyl)] after 7 d of degradation did not show any cytotoxic activity against L929 fibroblast cells. The polymer and its degradation products are non-toxic and biocompatible. The cloud point at 29°C (between room and body temperature) of this thermosensitive polydepsipeptide make it attractive for implants and other biomedical applications.

**Scheme 11:**
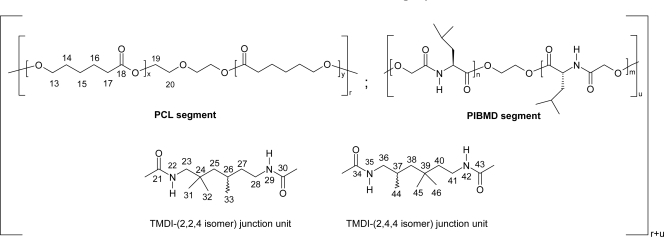
PCL-PIBMD multiblock copolymer structure.

Feng *et al*. synthesized multiblock copolymers based on oligodepsipeptides with shape-memory properties [94]. Biodegradable implant polymeric materials with shape-memory properties have been developed for application in biomedicine [95]. These polymers have the capability to change their shape when stimulated by changes in environmental factors such as temperature. Thermoplastic phase-segregated multiblock copolymers with polydepsipeptides and poly(ε-caprolactone) segments were synthesized via coupling of oligodepsipeptides and oligo(ε-caprolactone) diol (PCL-diol) using an aliphatic diisocyanate (Scheme 11). The multiblock copolymers showed good elastic properties at 25 and 75 °C and a shape-memory capability. An almost complete fixation of the mechanical deformation ε_m_ resulting in the temporary shape as well as quantitative recovery of the permanent shape in five cycles with a switching temperature *T*_sw_ around body temperature were observed. In hydrolytic degradation experiments at 37 °C in PBS buffer solution having a pH of 7.4 the multiblock copolymers showed a fast decrease of the molecular weight without induction period and changed from an elastic material to a brittle one in 21 d. The degradation of the multiblock copolymer showed a faster degradation of the poly(3-(*S*)-isobutylmorpholine-2,5-dione) domains in contrast to the PCL domains. As these materials combine shape-memory capability and degradability in terms of multifunctionality they have a high application potential for biomedical applications such as smart implants or medical devices.

The hydrophilicity and degradability were adjusted by introduction of hydrophilic block of polyethylene glycol (PEG) into polydepsipeptides. Block copolymers with poly(3-methylmorpholine-2,5-dione) (PMMD) and PEG blocks, PMMD-b-PEG-b-PMMD, were synthesized via ring-opening polymerization of 3-methylmorpholine-2,5-dione with amino-terminated PEG as the initiator at 140 °C within 10 h [96].

Four different types of polydepsipeptide-polyether block copolymers were synthesized via ring-opening polymerization of 3(*S*)-*sec*-butylmorpholine-2,5-dione or 3(*S*)-isobutylmorpholine-2,5-dione in the presence of poly(ethylene oxide) with one, two, three and four terminal OH-groups with stannous octoate as a catalyst, i.e., an AB block copolymer, an ABA block copolymer, an (A)_2_B star shaped block copolymer and an (A)_2_B(A)_2_ star shaped block copolymer, respectively, where A is a polydepsipeptide and B a PEO block (Scheme 11) [97–100]. The molar ratio of depsipeptide to PEO was varied to obtain copolymers with different weight fractions of polydepsipeptide blocks ranging from 47 to 97.5 wt%. The crystallinity of the PEO block decreased in the following order: AB > (A)_2_B > ABA > (A)_2_B(A)_2_. The hydrophilicity of the block copolymers increased greatly with increasing PEO content in the copolymers.

**Scheme 12:**
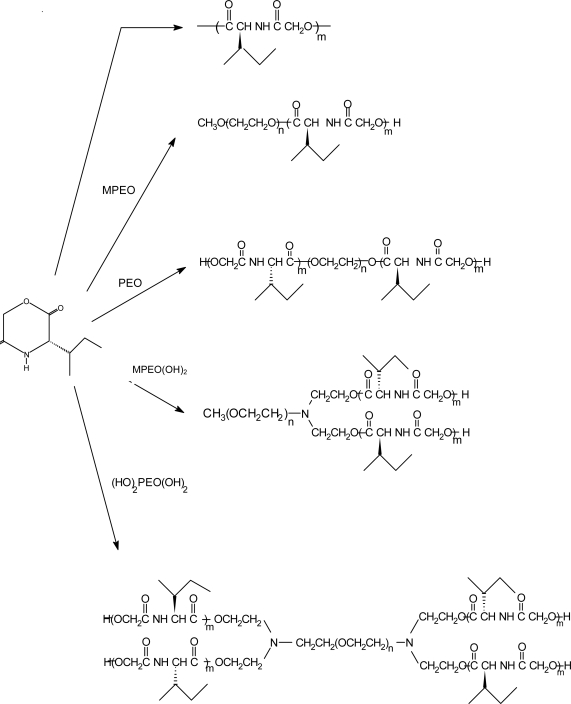
Reaction scheme of the preparation of polydepsipeptide-PEO block copolymers.

**Scheme 13:**
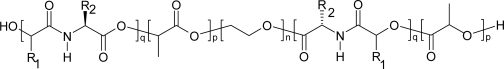
Block copolymers based on depsipeptides, DLLA or LLA and PEO.

ABA block copolymers were synthesized via ring-opening polymerization of morpholine-2,5-diones and DLLA or LLA in the presence of PEO8000 as an initiator and stannous octoate as a catalyst at 140 °C for 9 h (Scheme 12 and 13). *In vitro* degradation experiments revealed that the block copolymers based on depsipeptides, DLLA and PEO8000 lost their weight faster than the block copolymers based on depsipeptides, LLA and PEO. The weight loss rates depended on the weight fraction of PEO in the block copolymers and the starting molecular weight. The molecular weight decreased quickly, while the molecular weight distribution increased up to a polydispersity from 1.63 to 4.29 with increasing degradation time.

Ouchi *et al*. synthesized amphiphilic AB-type diblock copolymers composed of hydrophobic polylactide segment and hydrophilic polydepsipeptide segment with amino groups or carboxyl groups [101]. The morpholine-2,5-diones with protected functional side-chain groups such as cyclo[Glc-Lys(Z)] and cyclo[Glc-Asp(OBzl)] (Lys is lysine, Asp is aspartic acid, Z is benzyloxycarbonyl, and OBzl is benzyl) were first polymerized in tetrahydrofuran (THF) with potassium ethoxide as an initiator to obtain the corresponding protected polydepsipeptides. After the terminal hydroxyl groups of the protected polydepsipeptides were converted into the potassiumalcoholates as macroinitiators, L-lactide was polymerized in THF to obtain poly[Glc-Lys(Z)]-*block*-poly(l-lactide) and poly[Glc-Asp(OBzl)]-*block*-poly(l-lactide). Subsequent deprotection of Z and OBzl groups obtained the amphiphiles poly(Glc-Lys)-*block*-poly(l-lactide) and poly(Glc-Asp)-*block*-poly(l-lactide), respectively. These amphiphilic AB-type diblock copolymers of PLA and polydepsipeptides with amino or carboxyl groups formed core–shell polymeric micelles (50–100 nm in diameter) with chemically modifiable surfaces in aqueous solutions.

## 5. Polydepsipeptides and their copolymers used as biomaterials

The copolymers based on l-lactide and 6,6’-dimethylmorpholine-2,5-dione containing ester and amido functional groups in the backbone had lower glass transition temperatures and crystallinity compared to homopolymer PLLA. The adhesion of D1 mouse stem cells on copolymer films with low amido functional groups content was attenuated compared to pure PLLA [102]. The copolymers were nontoxic, but did not appear to induce high cell proliferation, might be extremely useful in regenerating tissues that mandate high mechanical integrity and low cell volume (e.g. cartilage or spinal disc).

The copolymer synthesized from l-lactide and morpholine-2,5-dione derivative with COOH functional groups was grafted with heparin by covalent binding using *N,N*-dicyclohexylcarbodiimide (DCC) as the coupling reagent [103]. The biodegradable copolymer was coated on the coronary artery stent. The coating polymer was completely degraded after implantation for 3 months. The heparin was controlled released during the degradation of the copolymers. No residual polymers were found in the surrounding tissue. No in-stent thrombosis and restenosis were observed in any stenting gmups, indicating good biocompatibility *in vivo*.

Amphiphilic ABA-type triblock copolymers of poly[LLA-*co*-(Glc-Leu)]-PEG- poly[LLA-*co*-(Glc-Leu)] were used to develop a biodegradable anti-adhesive membrane. The poly[LLA-*co*-(Glc-Leu)] was used as the hydrophobic segment A, and the PEG with Mn 10,000 and Mn 20,500 as the hydrophilic segment B. The degradation rate of the triblock copolymer films varied with changed in the molecular architecture, specifically, the molecular weight of the hydrophilic segment and the depsipeptide unit content in the A segment were more prominent [104]. The films were degraded and depleted gradually *in vivo* without inflammation.

The effects of charged groups on poly[(Glc-Asp)-*co*-LA] and poly[(Glc-Lys)-*co*-LA] film surfaces with respect to cell attachment and growth of mouse fibroblast L929 cells were investigated. The films with reactive surfaces had higher cell attachment ability than PLLA film [105]. During cell culture, the polydepsipeptide films exhibited higher degradation rates related to the depsipeptide content in the copolymers. The introduction of hydrophilic depsipeptide units in PLLA leaded to a decrease in crystallinity. The increase of the amorphous region and the hydrophilicity resulted in water molecules to infiltrate more easily, and thus a rapid hydrolytic degradation. Varying the depsipeptide content could control the degradation rates *in vivo*. These copolymers are expected to be applicable as degradable and chemically modifiable temporary scaffolds for tissue engineering in various situations.

3-Dimensional porous sponges as functional scaffolds for tissue regeneration were prepared from poly[(Glc-Asp)-*co*-LA] and poly[(Glc-Lys)-*co*-LA] materials by freeze-drying method [106]. Good cell growth was observed on the copolymer sponges. During cell culture, the copolymer sponges exhibited various degradation rates related to the depsipeptide content which is similar to polydepsipeptide films. Three-dimensional biodegradable polymer sponges with reactive surface, controllable degradation behavior and good cell growth are good candidate for scaffolds for tissue engineering.

Arginine-Glycine-Aspartic (RGD) peptides were chemical conjugated to the primary ε-amine groups of lysine components of diblock copolymer poly(lactic acid-co-lysine) (poly(LLA-co-Lys), 2.1 mol% lysine) [60,83,107]. RGD was the minimal cell-recognizable sequence in many extracellular matrix proteins and blood proteins [108]. Integrin αvβ3 is a receptor for the extracellular matrix proteins with exposed RGD tripeptide sequence and normally expressed at low levels on epithelial cells and mature endothelial cells but highly expressed on the activated endothelial cells in the neovasculature of tumors, including osteosarcomas, glioblastomas, melanomas, lung carcinomas, and breast cancer [109]. The overexpression of integrin αvβ3 during tumor growth, invasion, and metastasis presents an interesting drug-targeted method [110]. Therefore, the RGD-grafted diblock copolymer is expected to find application in drug carriers for tumor therapy or non-viral DNA carriers for gene therapy.

Sequential polydepsipeptides poly[(Ala)_n_-Glu(OEt)-Lac] were used as biodegradable carriers for drug delivery systems [111]. The *in vivo* degradation of these copolymers was strongly influenced by Ala units during subcutaneous implantation in the backs of male rats. Poly(Ala-Ala-Glu(OEt)-Lac) showed the highest degradability, in which 100% degradation was observed in 24 weeks implantation.

## 5. Conclusions

The polydepsipeptides with reactive carboxyl or amino groups as functional groups are suitable for covalently binding bioactive molecules to control cell interaction, such as immobilization of RGD peptides to enhance the cell attachment ability. Moreover, the microspheres prepared from copolymers and block copolymers containing functional (hydrophilic and ionic) groups exhibit more efficient entrapment of ionic substances by electrostatic interaction than PLLA microspheres. The microspheres showed controlled release of drugs and growth factors from the biodegradable matrix to promote rapid growth of cells and the regeneration of tissue. The degradation of polydepsipeptides is expected to reduce the local concentration of acid formed upon degradation as compared to polylactides which in certain cases might be advantageous for other biodegradable polyesters. Bioresorbable polymers based on polydepsipeptides could be used as biomaterials in drug controlled release, tissue engineering scaffolding, and shape-memory materials.

## Figures and Tables

**Table 1:** Ring-opening polymerization of 3(S)-isopropyl-morpholine-2,5-dione with/without lipase.

Entry	Lipasea)	wt%	Temp. in °C	Time in h	Conv.b)in %	Mnb)10^3^	Mw/Mnb)
1	PPL	1.5	100	72	3.2	6.1	1.10
2	PPL	5	100	72	26.2	10.4	1.05
3	PPL	10	100	72	53.6	11.0	1.08
4	PS	1.5	100	72	12.2	17.5	1.50
5	PS	4.7	100	168	73.8	12.5	3.33
6	PC	10	100	72	20.8	4.50	1.84
7	CR	10	100	72	46.3	3.50	1.99
8	Novo-435	10	100	72	5.6		
9	blank	0	100	168	0		

a) PPL: porcine pancreatic lipase, PS: lipase type XIII from *Pseudomonas species*, PC: lipase from *Pseudomonas cepacia*, CR: lipase type VII from *Candida rugosa* and Novo-435: lipase from *C. Antarctica* (Novozyme-435).

b) Determined by means of GPC using DMAc as eluent and polystyrene standards for calibration.
